# Mineral Intake and Depression: A Cross-Sectional Comparative Study Based on National Health and Nutrition Examination Surveys in Korea and the United States

**DOI:** 10.3390/nu17162593

**Published:** 2025-08-09

**Authors:** Jiwoo Kim, Inho Kim, Junhui Lee, Kyungwhan Jeon, Juseong Kang, Dongchan Lee, Sera Choi, HyunSoo Kim, Minkook Son

**Affiliations:** 1 Department of Physiology, College of Medicine, Dong-A University, Busan 49201, Republic of Korea; 2 New York Institute of Technology of Osteopathic Medicine, Old Westbury, NY 11568, USA; 3 Pusan National University of Medicine, Busan 46241, Republic of Korea; 4 Department of Psychiatry, College of Medicine, Dong-A University, Busan 49201, Republic of Korea; 5 Interdisciplinary Program, Department of Data Science Convergence, Dong-A University, Busan 49201, Republic of Korea

**Keywords:** minerals, depression, cross-sectional studies, Republic of Korea, United States

## Abstract

**Background/Objectives**: Depression is a major global health burden, and previous studies suggest that nutrient deficiencies may contribute to its development. However, research on mineral intake and depression, particularly sodium and potassium, is limited. We conducted a cross-sectional analysis of data from the National Health and Nutrition Examination Survey in Korea (KNHANES) and the United States (NHANES) to assess associations between various mineral intakes and depression in Korean and American adults. **Methods**: This cross-sectional study used the KNHANES and NHANES data. Seven minerals were analyzed: sodium, potassium, phosphorus, magnesium, iron, zinc, and calcium. Depression was defined as a Patient Health Questionnaire-9 score of 10 or higher. Associations between mineral intakes and depression were examined using a multivariable-adjusted logistic regression analysis. **Results**: In KNHANES, 537 participants (4.1%) exhibited depression, whereas 588 participants (6.2%) in NHANES experienced similar conditions. In addition, sodium, potassium, and phosphorus intake in KNHANES demonstrated inverse associations with depression, while in NHANES, potassium, iron, and zinc exhibited comparable trends. Subgroup analyses by sex, obesity status, and age revealed significant differences for several minerals. **Conclusions**: This study revealed significant associations between mineral intake and depression in both Korean and American adults. These findings suggest that adequate mineral intake may support mental health. Further research is needed to explore these relationships and underlying mechanisms.

## 1. Introduction

Depression is a common mood disorder characterized by persistent feelings of sadness, worthlessness, and hopelessness, and is anticipated to become one of the leading contributors to the global burden of disease in the future [1]. According to the Korea Disease Control and Prevention Agency’s Korea National Health and Nutrition Examination Survey (KNHANES) in 2022, about 5% of participants had experienced a depressive disorder [2]. Similarly, in the United States, data from their NHANES suggest that about 17.3 million adults aged 18 and over experienced at least one major depressive episode [3]. Moreover, many individuals with depression do not receive appropriate treatment. In South Korea, Park et al. found that more than two-thirds of participants (67.7%) with major depressive disorder (MDD) did not receive appropriate treatment [4]. According to the Anxiety and Depression Association of America, 28.7% of individuals screened positive for depression yet remained untreated [5]. Recent studies have found that a reluctance to seek professional help is associated with damaging interpersonal relationships and the stigmas of mental illness [6,7]. Additionally, the high recurrence rate of depression complicates its treatment and management. Hence, it is necessary to identify individuals with untreated depression, comprehend the factors associated with depression, and establish measures for its treatment.

Numerous studies have identified associations between nutrition and depression [8]. Minerals such as magnesium, zinc, and selenium affect cognitive performance and brain function, and their deficiency can contribute to mental disorders, including schizophrenia, dementia, anxiety, and insomnia [9,10,11,12]. However, few studies have examined the roles of sodium and potassium. Sodium is abundant in the diets of South Koreans and Americans, primarily as table salt. Potassium is present in nearly all foods, including fruits, vegetables, dairy, and meat. Although both minerals are essential, systematic analyses of their relationship with depression are limited. Therefore, a comprehensive evaluation of dietary mineral intake is warranted.

Understanding how mineral intake influences depression can inform evidence-based nutrition guidelines for preventing or treating depression and guide future research on nutrition and mental health by comparing data from South Korea and the United States. Given the cultural, lifestyle, and healthcare infrastructure differences between these countries, studies that compare data across these populations are scarce. Our research provides valuable insights and establishes a foundation for future investigations into the nutritional determinants of mental health. Therefore, this study aimed to assess the association between the intake of seven minerals (sodium, potassium, phosphorus, magnesium, iron, zinc, and calcium) and depression by analyzing data from KNHANES and NHANES among individuals aged 19 years or older who are depressed but untreated and those without depression and without treatment.

## 2. Materials and Methods

### 2.1. Datasets and Study Population

We used data from the KNHANES and NHANES. KNHANES, conducted by the Korea Disease Control and Prevention Agency, and NHANES, conducted by the National Center for Health Statistics (NCHS), are large-scale, nationally representative cross-sectional surveys. Both employ stratified, multistage, clustered probability sampling to represent noninstitutionalized civilian populations in Korea and the United States.

**Figure 1:**
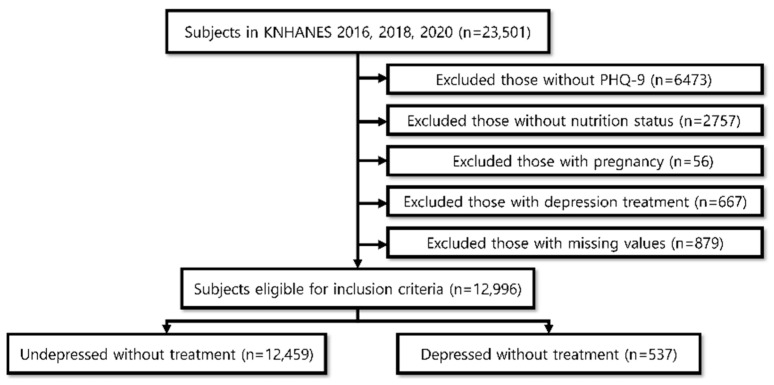
The flow of study populations in KNHANES.

We selected the KNHANES data from 2016, 2018, and 2020—years when the Patient Health Questionnaire-9 (PHQ-9) was administered. KNHANES has been conducted annually since 1998, surveying approximately 10,000 residents each year. It targets Korean citizens aged one year and older, ensuring a representative sample. KNHANES collects demographic, anthropometric, and clinical data via health interviews, physical examinations, and laboratory tests conducted by certified professionals. These data support health policy development and epidemiological research. Primary sampling units were defined by administrative districts and housing types, and secondary units were defined by households stratified for age, gender, and residence. Of the 23,501 participants aged 19 years and older who completed the PHQ-9, 12,996 were included in our analysis after excluding individuals with missing PHQ-9 data (*n* = 6473), missing nutrition data (*n* = 2757), pregnancy (*n* = 56), receiving treatment for depression (*n* = 667), and other missing values (*n* = 879) (Figure 1). Further details on KNHANES are available in the Korea Disease Control and Prevention Agency documentation and previous studies [13].

The NHANES data from 2013 to 2018 were selected because the International Classification of Diseases, 10th Revision, Clinical Modification (ICD-10-CM) coding of self-reported health conditions, which are used to calculate the Charlson Comorbidity Index (CCI), became available in 2013. Data after 2019 were excluded due to incompleteness resulting from the COVID-19 pandemic. The definition of depression in this study was based exclusively on PHQ-9 scores. NHANES has been conducted by the NCHS under the Centers for Disease Control and Prevention since the early 1960s, surveying approximately 10,000 individuals every two years to assess the health and nutritional status of the noninstitutionalized U.S. population. It collects demographic, socioeconomic, dietary, and health-related data through structured interviews and laboratory tests conducted at participants’ homes or at mobile examination centers. Like KNHANES, the primary sampling units of NHANES correspond to survey districts, while secondary sampling units consist of selected households. Of the 29,400 individuals aged 18 years and older who completed the PHQ-9 survey, 9547 were included in the analysis after applying the same exclusion criteria as KNHANES: missing PHQ-9 data (*n* = 13,826), missing nutrition data (*n* = 382), pregnancy (*n* = 117), receiving treatment for depression (*n* = 1335), and other missing values (*n* = 4173) (Figure S1). Further details on NHANES can be found on the NCHS documentation website [14].

### 2.2. Dietary Habits for Minerals

The dietary data from foods and beverages were collected using 24-h dietary recall methods in both the KNHANES and NHANES. For the KNHANES, mineral intake was calculated from the food intake frequency survey and assessed by 24-h recall. For the NHANES data, the United States Department of Agriculture Food and Nutrient Database for Dietary Studies was used to process total daily nutrient intake from foods and beverages. To investigate the association between mineral intake and depression, we included sodium, potassium, phosphorus, magnesium, iron, zinc, and calcium. All mineral intakes were expressed in milligrams.

### 2.3. Definition of Depression

The presence of depression was defined by the PHQ-9 data from KNHANES and NHANES. The PHQ-9 is a self-report survey designed to screen for MDD based on the Diagnostic and Statistical Manual of Mental Disorders, fifth edition [15]. It consists of nine items, each scored from 0 to 3 based on the frequency of symptoms experienced by the respondent over the past two weeks: 0 (not at all), 1 (several days), 2 (more than half the days), and 3 (nearly every day). PHQ-9 scores of 10 or higher were used as the threshold for identifying depression, following previous research demonstrating its sensitivity of 88% and specificity of 88% for identifying MDD [15].

### 2.4. Covariates

In this study, the covariates included demographic, socioeconomic, health, and nutritional status factors. Demographic and socioeconomic covariates included sex, age, race, income, education, marital status, and health insurance type. In the analysis of the KNHANES data, race was excluded due to the sample’s homogeneous ethnic characteristics. The income indicator used in KNHANES (code: incm) was originally collected as quartiles, and the family monthly poverty level index collected in NHANES (code: INDFMMPI) was used to derive income quartiles. Education level was categorized as having a bachelor’s degree or higher. Marital status was classified into six categories: “Married,” “Widowed,” “Divorced,” “Separated,” “Never married,” and “Living with partner.” Since all KNHANES participants were covered by National Health Insurance, this variable was not included in the KNHANES analysis.

Health-related factors were grouped into two categories: health screening variables and comorbidity variables. Health screening variables included body mass index (BMI), hemoglobin A1c levels, smoking status, alcohol consumption, and prevalence of aerobic physical activity. Smoking status was categorized as “Smoker,” “Ex-smoker,” and “Non-smoker,” while alcohol consumption was classified as drinkers and non-drinkers. Aerobic physical activity was assessed based on World Health Organization guidelines [16], which recommends 150 min of moderate-intensity or 75 min of vigorous-intensity aerobic exercise per week or an equivalent combination. Because NHANES did not directly provide this variable, it was derived by multiplying the daily physical activity duration by the number of active days per week.

Comorbidity variables included hypertension, diabetes, dyslipidemia, and the CCI [17]. Participants were classified as having hypertension if they met one or more of the following criteria: systolic blood pressure ≥ 140 mmHg, diastolic blood pressure ≥ 80 mmHg, a diagnosis of hypertension, prescription of antihypertensive medication, or use of such medication. Diabetes was defined by one or more of these criteria: hemoglobin A1c ≥ 6.5%, a diabetes diagnosis, or use of insulin or antidiabetic medications. Dyslipidemia was defined similarly, with participants classified as having dyslipidemia if they met one or more of the following: serum total cholesterol ≥ 240 mg/dL, a hypercholesterolemia diagnosis, prescription of cholesterol-lowering medication, or use of such medication. The CCI was calculated from relevant variables because neither KNHANES nor NHANES provided a direct score. In KNHANES, the CCI included six conditions: cerebrovascular accident, connective tissue disease, liver disease, renal disease, chronic pulmonary disease, and cancer. Because data on the severity of liver and renal diseases and on tumor metastasis were not available, these conditions were excluded. In NHANES, self-reported health issues coded with ICD-10-CM (codes RXDRSC1, RXDRSC2, RXDRSC3) were used to calculate the CCI. CCI scores were categorized as 0, 1, 2, and ≥3.

Nutritional factors included daily energy and macronutrient intake (carbohydrates, proteins, and fats). Both KNHANES and NHANES used a 24-h dietary recall method to gather nutrient information.

### 2.5. Statistical Analysis

Continuous variables in the baseline characteristics are presented as weighted means ± standard error, and categorical variables as weighted percentages ± standard error. Associations between mineral intake and depression were first examined using univariable logistic regression. Subsequently, multivariable-adjusted logistic regression models were constructed to control for potential confounding variables, including sex, age, income level, education, marital status, BMI, smoking status, alcohol consumption, regular exercise status, hypertension, diabetes, dyslipidemia, CCI, and energy intake (kcal). In addition, mineral intake levels were categorized into tertiles as categorical variables and further examined in relation to depression. Statistical analyses were conducted using SPSS Statistics for Windows, version 22.0 (IBM Corp., Armonk, NY, USA). The analyses incorporated the complex sampling design and the pre-calculated sample weights provided in the publicly available KNHANES and NHANES datasets, ensuring national representativeness.

A restricted cubic spline (RCS) analysis was conducted to assess potential nonlinear associations between continuous mineral intake and depression. RCS models were fitted separately for each mineral that showed a significant association in the multivariable-adjusted analysis. We used 4 knots at the 5th, 35th, 65th, and 95th percentiles according to each mineral and the mean value of the minerals as a reference value for each spline plot.

Subgroup analyses were performed to examine potential effect modification by sex, age group, and obesity status. Age was stratified into ≤65 years and >65 years. Obesity was defined using BMI cutoffs of 25 kg/m^2^ for Korean participants and 30 kg/m^2^ for American participants, respectively. For each subgroup, multivariable-adjusted logistic regression models were constructed with the same covariates as in the primary analysis.

### 2.6. Ethical Considerations

This study utilized publicly available, de-identified data from the KNHANES and NHANES. The KNHANES protocol was approved by the Institutional Review Board of the Korea Disease Control and Prevention Agency, and the NHANES protocol was approved by the Research Ethics Review Board of the National Center for Health Statistics. Written informed consent was obtained from all participants at the time of enrollment. As the datasets used in this analysis are publicly accessible and contain no personal identifiers, additional ethical approval and participant consent were not required for the present study. Further information regarding the datasets is available at the official websites of the KNHANES and NHANES.

## 3. Results

### 3.1. Baseline Characteristics of Study Population

The baseline characteristics of the KNHANES study population are shown in Table 1. A total of 12,996 participants were included, of whom 537 (4.1%) had depression. Significant differences between participants with and without depression were observed in sex, income level, education, marital status, smoking status, diabetes prevalence, and CCI scores. The depressive group included a higher proportion of females than males (60.7% vs. 39.3%). Participants with depression had lower monthly income, lower educational attainment, and were more likely to live alone. The mean PHQ-9 score was significantly higher in this group (13.43 vs. 1.77; *p* < 0.001). Furthermore, participants with depression had higher rates of smoking, higher diabetes prevalence, and higher CCI scores than those without depression. Mineral intake was generally lower in the depressive group than in the non-depressive group.

The baseline characteristics of the study population in NHANES are shown in Table S1. A total of 9547 participants were included; 588 (6.2%) exhibited depression. Significant differences between participants with and without depression were observed in sex, race, income level, insurance status, education, marital status, smoking status, regular exercise, diabetes, and CCI. In the depressive group, females outnumbered males (61.4% vs. 38.6%). Uninsured individuals and racial minorities were more prevalent than in participants without depression. Participants with depression tended to have lower monthly incomes, lower educational attainment, and higher rates of living alone. The mean PHQ-9 score was significantly higher in the depressive group (13.75 vs. 1.97; *p* < 0.001). Regular exercise was less common among participants with depression. Higher rates of smoking, diabetes, and elevated CCI scores were also observed. Furthermore, mineral intake, except for calcium, was generally lower in individuals with depression than in the non-depressive group.

### 3.2. Association Between Minerals and Depression

The association between mineral intake and depression is presented in Table 2. In the KNHANES data, univariable analysis showed a significant association between the intake of all minerals and depression. In the multivariable-adjusted analysis, sodium (adjusted odds ratio [OR] 0.89, 95% confidence interval [CI] 0.82–0.96; *p* = 0.004) and potassium (adjusted OR 0.87, 95% CI 0.77–0.99; *p* = 0.04) remained significantly associated, while phosphorus showed borderline significance (adjusted OR 0.64, 95% CI 0.41–1.00; *p* = 0.05).

In the NHANES data, univariable analysis indicated significant associations between the intake of all minerals, except calcium, and depression, with sodium showing borderline significance (Table 2). In the multivariable-adjusted analysis, potassium (adjusted OR 0.76, 95% CI 0.64–0.91; *p* = 0.003) and zinc (adjusted OR 0.83, 95% CI 0.70–0.99; *p* = 0.03) remained significantly associated, while iron showed borderline significance (adjusted OR 0.89, 95% CI 0.79–1.00; *p* = 0.05).

For minerals that showed significant associations, mineral intake levels were categorized into tertiles and further examined in relation to depression, as presented in Table S2.

**Figure 2:**
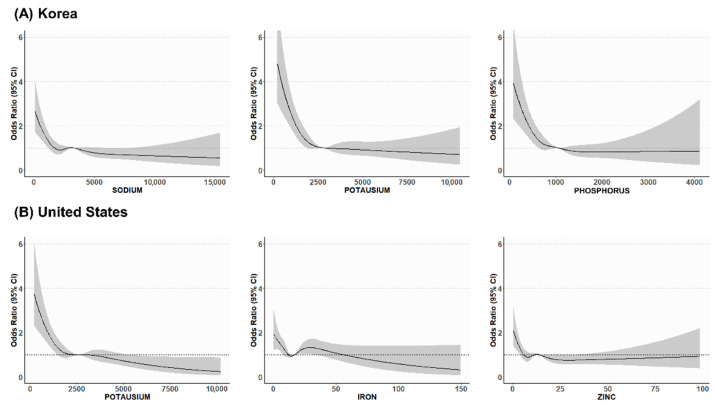
Restricted cubic spline curves according to minerals. The gray-shaded area surrounding the restricted cubic spline curves indicates the 95% confidence interval.

RCS analysis visualized the nonlinear relationship between mineral intake and depression (Figure 2). Among the minerals that retained significance in the multivariable-adjusted analysis, intake below a specific threshold was associated with a higher risk of depression; the remaining associations are presented in Figure S2.

### 3.3. Subgroup Analysis

**Figure 3:**
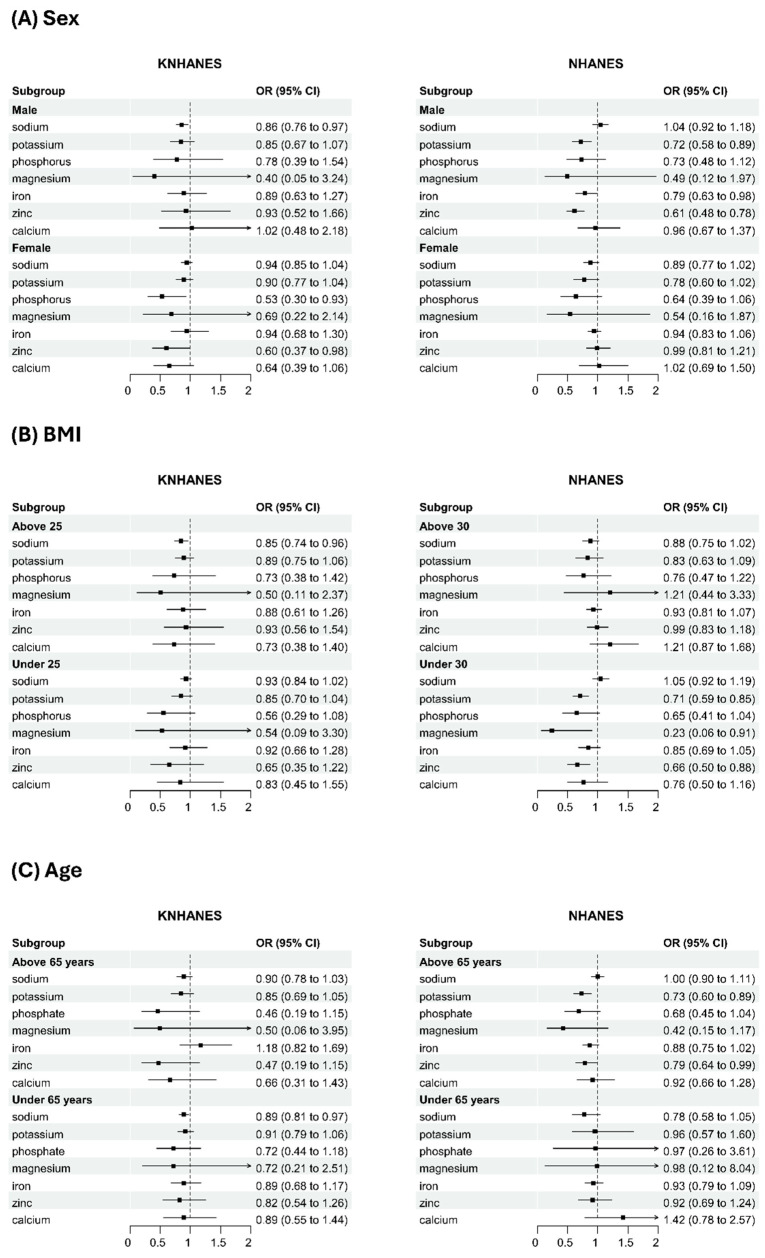
Subgroup analysis visualization.

The results of the subgroup analysis on the correlation between mineral intake and depression are presented in Figure 3. Certain minerals exhibited significant correlations with depression only within specific subgroups. In the sex-stratified analysis, for instance, among Korean males, sodium intake showed a significant negative correlation with depression (adjusted OR 0.86, 95% CI 0.76–0.97), while among Korean females, phosphorus intake demonstrated a similar significant negative correlation (adjusted OR 0.53, 95% CI 0.30–0.93). In American males, potassium, iron, and zinc intake were significantly negatively correlated, whereas no significant correlations were observed in American females. In a BMI-stratified analysis, sodium intake showed a significant negative correlation in the Korean obese group, while potassium, magnesium, and zinc intake showed a significant negative correlation in the American non-obese group. In the age-stratified analysis, sodium intake was significantly negatively correlated in Koreans aged 65 or younger, while potassium intake was notably negatively correlated in Americans above 65.

## 4. Discussion

This study analyzed data from the KNHANES and NHANES to investigate the association between intake of seven mineral types and depression. Using large, representative samples from Korea and the United States, we found inverse associations between intake of specific minerals and depression. In KNHANES, sodium, potassium, and phosphorus intake were inversely associated with depression, while in NHANES, potassium, iron, and zinc showed similar negative associations. No association between magnesium or calcium intake and depression was observed in either country. Several studies have reported links between these minerals and their effects on neuronal activity or mental health.

### 4.1. Sodium

Sodium intake was inversely associated with depression in KNHANES. One plausible mechanism involves activation of ventral pallidum neurons due to sodium depletion and stimulation of the trigeminal nerve by salt intake. Low serum sodium levels have been linked to depression [18], and correction of hyponatremia can improve these symptoms [19]. A second mechanism involves the renin–angiotensin–aldosterone system. Our results align with animal studies showing that sodium deficiency induces depressive behaviors reversible by sodium intake [18]. Previous studies suggest that angiotensin-converting enzyme inhibitors used for hypertension may alleviate depression by modulating this system [20].

### 4.2. Potassium

A significant inverse correlation between dietary potassium intake and depression aligns with previous evidence identifying potassium intake as a key predictor of depression [21]. One potential mechanism links insufficient potassium to disrupted neuronal excitability and neurotransmitter balance, thereby increasing mood disorder susceptibility. This relationship is further supported by the role of potassium channels in mood regulation [22].

### 4.3. Phosphorus

Although a direct link between dietary phosphorus intake and depression has not yet been established, several studies support a connection between low phosphate levels and mood disorders. Lower serum phosphate has been significantly associated with anxiety and somatic symptoms in patients with depression [23], as well as in panic disorder [24]. Another perspective involves adenosine triphosphate (ATP) synthesis and release: hypophosphatemia reduces ATP production, and insufficient ATP release from astrocytes may contribute to depression [25,26].

### 4.4. Iron

Iron deficiency or low iron intake is linked to neural alterations that can contribute to depression, particularly via effects on γ-aminobutyric acid (GABA) [27]. In rats, brain iron deficiency alters GABA metabolism. In humans, patients with MDD have significantly lower GABA levels than controls [28]. Positive allosteric modulation of GABA(A) receptors has demonstrated antidepressant activity in animal models. GABAergic agents can function similarly to tricyclic antidepressants in forced-swim-induced depression [29].

### 4.5. Zinc

Zinc release in the brain modulates Zn^2+^ signaling in the hippocampus and may contribute to depression pathophysiology [30]. Scewzyk et al. reported that zinc deficiency reduces synaptic zinc levels [31]. Synaptic zinc modulates various glutamate receptors, including the α-amino-3-hydroxy-5-methyl-4-isoxazolepropionic acid receptor, which regulates zinc homeostasis in the hippocampus [32]. Disrupted hippocampal development due to zinc deficiency may also lead to depression, as patients with MDD exhibit reduced hippocampal volume [33].

Although higher mineral intake generally correlates with reduced depression, the strength of this negative correlation varied by mineral and population. In KNHANES, sodium, potassium, and phosphorus showed significant negative correlations; in NHANES, potassium, iron, and zinc were significant. Notably, potassium was the only mineral consistently correlated across both datasets, underscoring its potential role in mental health. These disparities may reflect differences in average mineral intake between Korea and the United States [13,34]. For example, potassium intake was comparable (2.60 g/day in Korea vs. 2.65 g/day in the United States), consistent with its association with reduced depressive symptoms. In contrast, sodium showed a significant association only in Korea, likely reflecting its substantially higher baseline intake (~4600 mg/day), with fermented vegetables, soups, and stews as major contributors [35]. Such foods provide sodium in a distinct nutritional context compared with the processed food sources more typical in the United States, and traditional dietary habits create considerable within-population variation in sodium intake. In comparison, higher iron and zinc intakes in the United States (iron: 19.17 mg/day vs. 9.61 mg/day; zinc: 16.45 mg/day vs. 10.16 mg/day) may account for their significance in NHANES. In addition to differences in mean intake, variations in dietary sources [36,37] and nutrient bioavailability [38] are also important. In the United States, red meat is the predominant source of iron [36], providing highly bioavailable heme iron [38], whereas in Korea, iron is mainly derived from plant-based and seafood sources [37], which supply less bioavailable non-heme iron, further inhibited by phytates and polyphenols [38]. Similarly, zinc absorption is enhanced by animal proteins but inhibited by phytate-rich cereals and legumes, which are more common in the Korean diet [39]. Collectively, differences in baseline intake levels, dietary sources, and nutrient bioavailability may help explain the observed population-specific associations, consistent with previous reports linking dietary patterns to depressive symptoms [40,41].

This study had several strengths and limitations. Its major strength was the leveraging of data from two countries—Korea and the United States—providing a broader perspective on mineral intake–depression relationships. The inclusion of seven minerals enabled insights into potential underlying mechanisms. Additionally, subgroup analyses stratified by gender, BMI, and age identified factors influencing both mineral intake and depression.

However, the cross-sectional design of the study cannot infer causal inferences, and dietary habits might be a consequence rather than a cause of depression, suggesting the potential for reverse causation. Another limitation is the lack of biological indicators. Dietary intake was assessed via a 24-h recall, relying solely on participants’ memory. This approach is prone to memory bias and may yield inaccurate intake estimates. Given that memory impairment commonly occurs in depression, these inaccuracies may be exacerbated, contributing to inconsistent associations. Finally, the PHQ-9 is a self-report measure and may be subject to social desirability bias and recall errors.

## 5. Conclusions

Overall, these findings support integrating nutritional guidance into public health strategies aimed at preventing and managing depressive symptoms. Significant associations between specific minerals and depression suggest that dietary interventions may play a crucial role in mental health care. However, further research is needed to validate these findings and elucidate the underlying mechanisms. Longitudinal studies, notably, could help establish causality and provide deeper insights into how mineral intake influences mental health over time. By laying a strong foundation for future investigations, this study contributes to public health efforts aimed at leveraging the connection between diet and mental well-being.

## Figures and Tables

**Table 1:** Baseline characteristics of the study population in KNHANES.

	Depression		
Variables	Without (*n* = 12,459)	With (*n* = 537)	*p*-Value
Demographic			
Sex (%)			<0.001
Male	51.8 (0.5)	39.3 (2.6)	
Female	48.2 (0.5)	60.7 (2.6)	
Age (years)	46.94 (0.25)	45.97 (0.95)	0.30
Income level (%)			<0.001
1 (low)	23.1 (0.7)	35.7 (2.6)	
2	24.5 (0.6)	27.7 (2.1)	
3	25.7 (0.6)	20.3 (2.0)	
4 (high)	26.7 (0.9)	16.3 (2.0)	
Education (%)			0.002
Under college	45.2 (0.7)	53.2 (2.6)	
Above college	54.8 (0.9)	46.8 (2.6)	
Marriage status (%)			<0.001
Married	66.2 (0.7)	47.0 (2.7)	
Separated	0.6 (0.1)	1.0 (0.4)	
Widowed	5.3 (0.2)	9.8 (1.2)	
Divorced	3.5 (0.2)	9.2 (1.3)	
Never	24.4 (0.7)	33.0 (2.7)	
Health screening			
PHQ-9	1.77(0.03)	13.43 (0.19)	<0.001
Body mass index (cm/m^2^)	24.05 (0.04)	24.52 (0.23)	0.04
Waist size (cm)	83.1 (0.12)	84.23 (0.61)	0.07
Systolic blood pressure (mmHg)	117.6 (0.21)	117.65 (1.0)	0.96
Diastolic blood pressure (mmHg)	76.20 (0.12)	75.28 (0.57)	0.11
Total cholesterol (mg/dL)	191.81 (0.43)	190.47 (1.98)	0.51
HbA1c (%)	5.67 (0.01)	5.78 (0.06)	0.05
Smoking status (%)			<0.001
Non-smoker	59.9 (0.5)	52.9 (2.6)	
Ex-smoker	20.5 (0.4)	15.6 (1.8)	
Smoker	19.6 (0.5)	31.5 (2.5)	
Alcohol drinking (%)	77.2 (0.5)	73.0 (2.1)	0.04
Regular exercise (%)	47.1 (0.6)	44.0 (2.7)	0.27
Comorbidities			
Hypertension (%)	27.2 (0.5)	29.4 (2.4)	0.18
Diabetes (%)	10.5 (0.3)	18.6 (1.9)	<0.001
Dyslipidemia (%)	19.9 (0.4)	22.8 (2.1)	0.16
Charlson comorbidity index (%)			<0.001
0	87.8 (0.3)	79.4 (1.9)	
1	7.0 (0.3)	11.8 (1.4)	
2	4.6 (0.2)	6.1 (1.0)	
3	0.7 (0.1)	2.6 (0.7)	

**Table 2:** Association between mineral intake and depression.

All Subjects	Odds Ratio (95% Confidence Intervals)			
	Crude	*p*-Value	Adjusted *	*p*-Value
KNHANES				
Sodium (per 1000 mg)	0.87 (0.82, 0.93)	<0.001	0.89 (0.82, 0.96)	0.004
Potassium (per 1000 mg)	0.82 (0.74, 0.91)	<0.001	0.87 (0.77, 0.99)	0.04
Calcium (per 1000 mg)	0.56 (0.37, 0.85)	0.007	0.79 (0.51, 1.25)	0.31
Magnesium (per 1000 mg)	0.22 (0.09, 0.56)	0.002	0.53 (0.17, 1.69)	0.28
Phosphorus (per 1000 mg)	0.61 (0.46, 0.80)	<0.001	0.64 (0.41, 1.00)	0.05
Iron (per 10 mg)	0.74 (0.59, 0.92)	0.006	0.90 (0.71, 1.15)	0.40
Zinc (per 10 mg)	0.65 (0.49, 0.86)	0.003	0.77 (0.51, 1.16)	0.21
NHANES				
Sodium (per 1000 mg)	0.92 (0.84, 1.00)	0.05	0.98 (0.89, 1.08)	0.69
Potassium (per 1000 mg)	0.74 (0.64, 0.85)	<0.001	0.76 (0.64, 0.91)	0.003
Calcium (per 1000 mg)	0.81 (0.63, 1.05)	0.11	0.98 (0.75, 1.29)	0.88
Magnesium (per 1000 mg)	0.18 (0.07, 0.46)	0.001	0.51 (0.21, 1.21)	0.12
Phosphorus (per 1000 mg)	0.68 (0.52, 0.89)	0.006	0.71 (0.49, 1.04)	0.08
Iron (per 10 mg)	0.82 (0.72, 0.94)	0.006	0.89 (0.79, 1.00)	0.05
Zinc (per 10 mg)	0.73 (0.61, 0.88)	0.001	0.83 (0.70, 0.99)	0.03

* Adjusted for sex, age, income level, education, marriage status, body mass index, smoking, alcohol drinking, regular exercise status, hypertension, diabetes, dyslipidemia, Charlson comorbidity index, and kcal consumption.

## Data Availability

This study utilized publicly available, de-identified data from the KNHANES “https://knhanes.kdca.go.kr/knhanes/eng/main.do” (accessed on 2 July 2025) and NHANES “https://www.cdc.gov/nchs/nhanes/index.html” (accessed on 2 July 2025).

## Supplementary Materials

The following supporting information can be downloaded at: https://www.mdpi.com/article/10.3390/nu17162593/s1. Figure S1: The flow of study populations in NHANES; Figure S2: Restricted cubic spline curves according to minerals without significance; Table S1: Baseline characteristics of the study population in NHANES; Table S2: Association between mineral intake (as a categorical variable) and depression.

## References

[1] Mathers C.D., Loncar D., Samet J. (2006). Projections of global mortality and burden of disease from 2002 to 2030. PLoS Med..

[2] Korea Disease Control and Prevention Agency (2023). The 6th Korea National Health and Nutrition Examination Survey. 2022 National Health Statistics Reports.

[3] Anxiety & Depression Association of America (2024). What Is Depression?.

[4] Park S., Cho M.J., Bae J.N., Chang S.M., Jeon H.J., Hahm B.-J., Son J.-W., Kim S.G., Bae A., Hong J.P. (2012). Comparison of treated and untreated major depressive disorder in a nationwide sample of korean adults. Community Ment. Health J..

[5] Olfson M., Blanco C., Marcus S.C. (2016). Treatment of Adult Depression in the United States. JAMA Intern. Med..

[6] Ramana R., Paykel E.S., Cooper Z., Hayhurst H., Saxty M., Surtees P.G. (1995). Remission and relapse in major depression: A two-year prospective follow-up study. Psychol. Med..

[7] Yoshikawa E., Taniguchi T., Nakamura-Taira N., Ishiguro S., Matsumura H. (2017). Factors associated with unwillingness to seek professional help for depression: A web-based survey. BMC Res. Notes.

[8] Harbottle L., Schonfelder N. (2008). Nutrition and depression: A review of the evidence. J. Ment. Health.

[9] Janka Z. (2019). [Tracing trace elements in mental functions]. Ideggyogy. Szle..

[10] Kirkland A.E., Sarlo G.L., Holton K.F. (2018). The Role of Magnesium in Neurological Disorders. Nutrients.

[11] Lo K., Liu Q., Madsen T., Rapp S., Chen J.-C., Neuhouser M., Shadyab A., Pal L., Lin X., Shumaker S. (2019). Relations of magnesium intake to cognitive impairment and dementia among participants in the Women’s Health Initiative Memory Study: A prospective cohort study. BMJ Open.

[12] Tao M., Liu J., Cervantes D. (2022). Association between magnesium intake and cognition in US older adults: National Health and Nutrition Examination Survey (NHANES) 2011 to 2014. Alzheimer’s Dement. Transl. Res. Clin. Interv..

[13] Kweon S., Kim Y., Jang M.-J., Kim Y., Kim K., Choi S., Chun C., Khang Y.-H., Oh K. (2014). Data Resource Profile: The Korea National Health and Nutrition Examination Survey (KNHANES). Int. J. Epidemiol..

[14] National Health and Nutrition Examination Survey (2024). About NHANES. https://www.cdc.gov/nchs/nhanes/about/index.html.

[15] Kroenke K., Spitzer R.L., Williams J.B. (2001). The PHQ-9: Validity of a brief depression severity measure. J. Gen. Intern. Med..

[16] World Health Organization (2010). WHO Guidelines Approved by the Guidelines Review Committee. Global Recommen-Dations on Physical Activity for Health.

[17] Quan H., Li B., Couris C.M., Fushimi K., Graham P., Hider P., Januel J.-M., Sundararajan V. (2011). Updating and validating the Charlson Comorbidity Index and score for risk adjustment in hospital discharge abstracts using data from 6 countries. Am. J. Epidemiol..

[18] Grippo A.J., Moffitt J.A., Beltz T.G., Johnson A.K. (2006). Reduced hedonic behavior and altered cardiovascular function induced by mild sodium depletion in rats. Behav. Neurosci..

[19] Sawant N.S., Parkar S.R., Rupani K., Bansal H., Singh S. (2019). Hyponatremia misdiagnosed as depression. Ann. Indian Psychiatry.

[20] Murck H., Schüssler P., Steiger A. (2012). Renin-angiotensin-aldosterone system: The forgotten stress hormone system: Relationship to depression and sleep. Pharmacopsychiatry.

[21] Huang A.A., Huang S.Y. (2023). Exploring Depression and nutritional covariates amongst US adults using Shapely additive explanations. Health Sci. Rep..

[22] Cui Y., Yang Y., Ni Z., Dong Y., Cai G., Foncelle A., Ma S., Sang K., Tang S., Li Y. (2018). Astroglial Kir4.1 in the lateral habenula drives neuronal bursts in depression. Nature.

[23] Maddock R.J., Moses J.A., Roth W.T., King R., Murchison A., Berger P.A. (1987). Serum phosphate and anxiety in major depression. Psychiatry Res..

[24] Pérez-Costillas L., Montes M.R., Martínez-Ortega J.M., Carretero M.D., Gutiérrez-Rojas L., Gurpegui M. (2013). Phosphate levels as a possible state marker in panic disorder: Preliminary study of a feasible laboratory measure for routine clinical practice. J. Psychiatr. Res..

[25] Pesta D.H., Tsirigotis D.N., Befroy D.E., Caballero D., Jurczak M.J., Rahimi Y., Cline G.W., Dufour S., Birkenfeld A.L., Rothman D.L. (2016). Hypophosphatemia promotes lower rates of muscle ATP synthesis. FASEB J. Off. Publ. Fed. Am. Soc. Exp. Biol..

[26] Wang K., Huang S., Fu D., Yang X., Ma L., Zhang T., Zhao W., Deng D., Ding Y., Zhang Y. (2024). The neurobiological mechanisms and therapeutic prospect of extracellular ATP in depression. CNS Neurosci. Ther..

[27] Ward K.L., Tkac I., Jing Y., Felt B., Beard J., Connor J., Schallert T., Georgieff M.K., Rao R. (2007). Gestational and lactational iron deficiency alters the developing striatal metabolome and associated behaviors in young rats. J. Nutr..

[28] Godfrey K.E., Gardner A.C., Kwon S., Chea W., Muthukumaraswamy S.D. (2018). Differences in excitatory and inhibitory neurotransmitter levels between depressed patients and healthy controls: A systematic review and meta-analysis. J. Psychiatr. Res..

[29] Lloyd K., Morselli P., Depoortere H., Fournier V., Zivkovic B., Scatton B., Broekkamp C., Worms P., Bartholini G. (1983). The potential use of GABA agonists in psychiatric disorders: Evidence from studies with progabide in animal models and clinical trials. Pharmacol. Biochem. Behav..

[30] Takeda A. (2011). Zinc Signaling in the hippocampus and its relation to pathogenesis of depression. Mol. Neurobiol..

[31] Szewczyk B., Kubera M., Nowak G. (2011). The role of zinc in neurodegenerative inflammatory pathways in depression. Prog. Neuro-Psychopharmacol. Biol. Psychiatry.

[32] Takeda A., Sakurada N., Ando M., Kanno S., Oku N. (2009). Facilitation of zinc influx via AMPA/kainate receptor activation in the hippocampus. Neurochem. Int..

[33] Sheline Y.I., Wang P.W., Gado M.H., Csernansky J.G., Vannier M.W. (1996). Hippocampal atrophy in recurrent major depression. Proc. Natl. Acad. Sci. USA.

[34] Yan X., Wang X., Zhang J., Ming Z., Zhang C., Ma P., Liu Q., Xu Y., Cheng L., Pang X. (2024). National trends in nine key minerals intake (quantity and source) among U.S. adults, 1999 to March 2020. Nutr. J..

[35] Yon M., Lee Y., Kim D., Lee J., Koh E., Nam E., Shin H., Kang B.-W., Kim J.W., Heo S. (2011). Major Sources of Sodium Intake of the Korean Population at Prepared Dish Level: Based on the Korea National Health and Nutrition Examination Survey 2008–2009. Korean J. Community Nutr..

[36] Hooda J., Shah A., Zhang L. (2014). Heme, an Essential Nutrient from Dietary Proteins, Critically Impacts Diverse Physiological and Pathological Processes. Nutrients.

[37] Choi H.-J., Lee H.-J., Jang H.B., Park J.Y., Kang J.-H., Park K.-H., Song J. (2011). Effects of Maternal Education on Diet, Anemia, and Iron Deficiency in Korean School-Aged Children. BMC Public Health.

[38] Zimmermann M.B., Hurrell R.F. (2007). Nutritional iron deficiency. Lancet.

[39] Gibson R.S., Perlas L., Hotz C. (2006). Improving the Bioavailability of Nutrients in Plant Foods at the Household Level. Proc. Nutr. Soc..

[40] Li Y., Lv M.-R., Wei Y.-J., Sun L., Zhang J.-X., Zhang H.-G., Li B. (2017). Dietary patterns and depression risk: A meta-analysis. Psychiatry Res..

[41] Marx W., Lane M., Hockey M., Aslam H., Berk M., Walder K., Borsini A., Firth J., Pariante C.M., Berding K. (2021). Diet and depression: Exploring the biological mechanisms of action. Mol. Psychiatry.

