# Supplementary material for: Mineral Intake and Depression: A Cross-Sectional Comparative Study Based on National Health and Nutrition Examination Surveys in Korea and the United States
Source: Nutrients. 2025 Aug 9;17(16):2593. doi: 10.3390/nu17162593 (PMC12389228; doi:10.3390/nu17162593)

Table S1. Baseline characteristics of the study population in NHANES.

Variables	Depression<br>Without<br>(n = 12459)	 With<br>(n = 537)	P-value
**Demographic**			
Sex (%)			<0.001
Male	53.3 (0.6)	38.6 (2.6)	
Female	46.7 (0.6)	61.4 (2.6)	
Age (years)	47.03 (0.39)	45.3 (1.04)	0.088
Race			0.029
Mexican American	9.0(1.1)	7.9(1.3)	
Non-Hispanic Black	10.5(1.0)	12.4(1.4)	
Non-Hispanic White	66.2(2.0)	60.1(3.0)	
Other HIspanic	6.0(0.7)	8.1(1.2)	
Other race	8.3(0.6)	11.5(1.8)	
Income level (%)			<0.001
1 (low)	16.0 (0.9)	35.4 (3.0)	
2	19.2 (0.8)	25.4 (2.7)	
3	27.3 (0.8)	23.9 (2.1)	
4 (high)	37.4 (1.6)	15.2 (23.1)	
Insurance	85.0(0.9)	73.6(3.2)	<0.001
Education (%)			<0.001
Under college	34.8 (1.3)	47.5 (2.9)	
Above college	65.2 (1.3)	52.5 (2.9)	
Marriage status (%)			<0.001
Married	57.9 (1.2)	33.9 (2.9)	
Separated	2.0 (0.2)	5.5 (1.0)	
Widowed	4.3 (0.3)	7.5 (1.5)	
Divorced	9.4 (0.4)	16.8 (1.9)	
Living with partner	8.4(0.5)	12.8(1.7)	
Never	18.0 (0.8)	23.5 (2.3)	
**Health screening**			
PHQ-9	1.97(0.38)	13.75 (0.20)	<0.001
Body mass index (cm/m^2)	29.18 (0.15)	30.42 (0.50)	0.02
Systolic blood pressure (mmHg)	122.40 (0.30)	122.17 (0.95)	0.81
Diastolic blood pressure (mmHg)	70.93 (0.32)	70.80 (0.61)	0.85
Total cholesterol (mg/dL)	189..79 (0.85)	192.40 (2.90)	0.34
HbA1c (%)	5.63 (0.02)	5.73 (0.04)	0.05
Smoking status (%)			<0.001
Non-smoker	58.6 (0.9)	39.2 (3.5)	
Ex-smoker	25.3 (0.8)	24.1 (2.5)	
Smoker	16.1 (0.7)	36.7 (3.2)	
Alcohol drinking (%)	76.9 (1.1)	74.7 (2.6)	0.36
Regular exercise (%)	65.7 (0.7)	58.6 (2.7)	0.01
**Comorbidities**			
Hypertension (%)	48.7 (0.8)	51.5 (3.2)	0.44
Diabetes (%)	12.0 (0.5)	15.7 (1.6)	0.03
Dyslipidemia (%)	40.2 (0.8)	39.3 (2.8)	0.74
Charlson comorbidity index (%)			0.009
0	91.1 (0.4)	89.6 (1.5)	
1	6.5 (0.3)	6.6 (1.1)	
2	1.8 (0.2)	1.3 (0.3)	
3	0.6 (0.1)	2.5 (1.0)	

Table S2. Association between mineral intake (as a categorical variable) and depression.

All subjects		Odds ratio (95% confidence intervals)			
		Crude	P-value	Adjusted*	P-value
**KNHANES**					
Sodium	1st tertile	1.00 (reference)		1.00 (reference)	
	2nd tertile	0.74 (0.60, 0.91)	0.004	0.87 (0.70, 1.08)	0.2
	3rd tertile	0.58 (0.47, 0.72)	<0.001	0.75 (0.57, 0.99)	0.041
Potassium	1st tertile	1.00 (reference)		1.00 (reference)	
	2nd tertile	0.59 (0.48, 0.72)	<0.001	0.67 (0.53, 0.83)	<0.001
	3rd tertile	0.50 (0.40, 0.62)	<0.001	0.61 (0.46, 0.80)	<0.001
Phosphorus	1st tertile	1.00 (reference)		1.00 (reference)	
	2nd tertile	0.63 (0.52, 0.78)	<0.001	0.77 (0.61, 0.97)	0.026
	3rd tertile	0.54 (0.43, 0.67)	<0.001	0.69 (0.51, 0.94)	0.019
**NHANES**					
Potassium	1st tertile	1.00 (reference)		1.00 (reference)	
	2nd tertile	0.65 (0.53, 0.79)	<0.001	0.75 (0.61, 0.94)	0.011
	3rd tertile	0.61 (0.50, 0.75)	<0.001	0.74 (0.56, 0.97)	0.030
Iron	1st tertile	1.00 (reference)		1.00 (reference)	
	2nd tertile	0.62 (0.50, 0.76)	<0.001	0.68 (0.55, 0.85)	<0.001
	3rd tertile	0.69 (0.56, 0.84)	<0.001	0.79 (0.62, 1.00)	0.05
Zinc	1st tertile	1.00 (reference)		1.00 (reference)	
	2nd tertile	0.75 (0.61, 0.91)	0.003	0.83 (0.67, 1.03)	0.10
	3rd tertile	0.56 (0.46, 0.69)	<0.001	0.66 (0.52, 0.85)	0.001

*Adjusted for sex, age, income level, education, marriage status, body mass index, smoking, alcohol drinking, regular exercise status, hypertension, diabetes, dyslipidemia, Charlson comorbidity index, and kcal consumption.

Figure S1. The flow of study populations in NHANES.

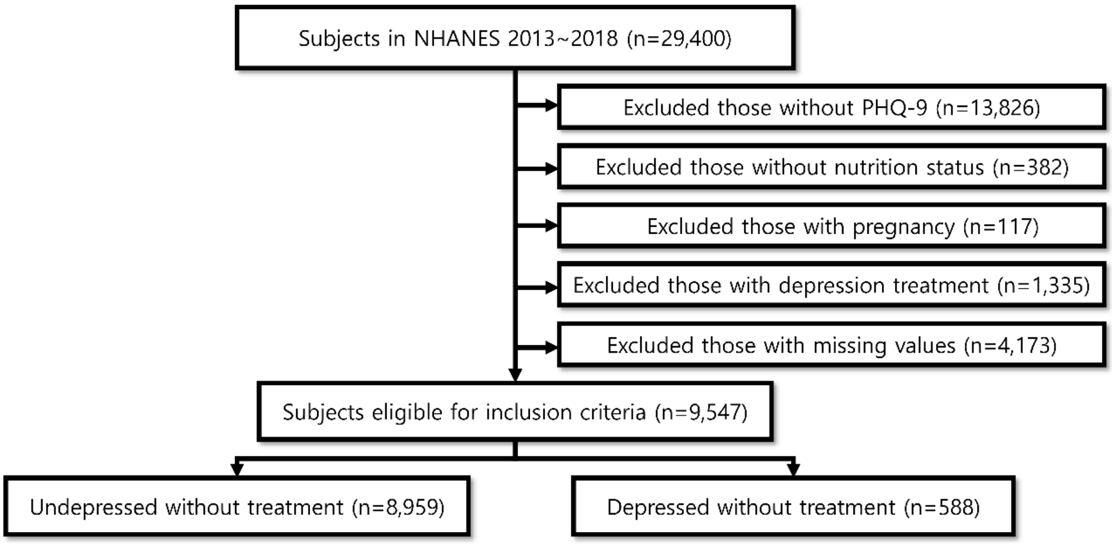

*NHANES, National Health and Nutrition Examination Survey.

Figure S2. Restricted cubic spline curves according to minerals without significance.

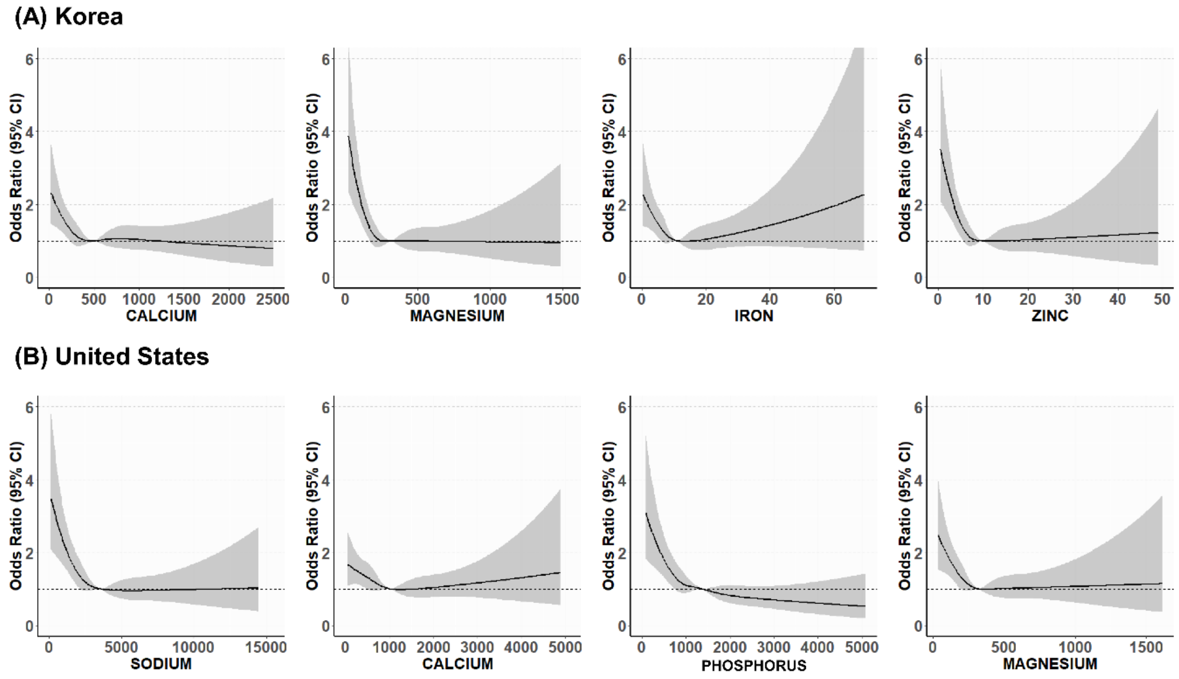

Supplement: Supplementary file 1 [file nutrients-17-02593-s001.zip › nutrients-3766401-supplementary.pdf]
